# Mechanical complications and radiographic outcomes following sacral-only versus spinopelvic fixation in long-segment fusion terminating at the sacrum: a retrospective cohort study

**DOI:** 10.1186/s12891-026-10225-0

**Published:** 2026-07-22

**Authors:** Christian Riediger, Mark Ferl, Christoph H. Lohmann, Agnieszka Halm-Pozniak, Maria Schönrogge

**Affiliations:** Department of Orthopaedics, University Hospital Magdeburg, Otto-von-Guericke- University Magdeburg, Magdeburg, Germany

**Keywords:** Spinal fusion, Spinopelvic fixation, S2-alar-iliac screws, Screw loosening, Sagittal alignment, Pseudarthrosis, Treatment outcome

## Abstract

**Purpose:**

Long-segment fusion terminating at the sacrum is associated with a substantial risk of distal mechanical complications, including screw loosening and pseudarthrosis. Whether spinopelvic fixation is associated with improved construct durability and maintenance of sagittal alignment compared with sacral-only fixation remains clinically relevant.

**Methods:**

This retrospective single-center cohort study included 100 adult patients undergoing posterior long-segment fusion involving four or more motion segments terminating at the sacrum between 2000 and 2024. Patients were stratified into sacral-only fixation and spinopelvic fixation groups. Radiographic outcomes included screw loosening, pseudarthrosis, sagittal vertical axis (SVA), and pelvic incidence–lumbar lordosis (PI–LL) mismatch. Clinical outcomes were assessed using the Oswestry Disability Index (ODI) over a standardized two-year follow-up.

**Results:**

Baseline characteristics were comparable between groups, although anterior lumbar interbody fusion (ALIF) at L5–S1 was performed more frequently in the spinopelvic fixation group (46% vs. 14%, *p* < 0.001). Sacral-only fixation was associated with higher rates of pseudarthrosis (48% vs. 12%, *p* < 0.001) and clinically relevant screw loosening (34% vs. 6%, *p* = 0.004). Spinopelvic fixation was associated with better maintenance of PI–LL mismatch during follow-up, whereas final SVA values were comparable between groups. Both groups demonstrated clinically meaningful functional improvement, although recovery occurred earlier in the spinopelvic fixation cohort.

**Conclusion:**

Spinopelvic fixation was associated with lower rates of distal mechanical complications and improved maintenance of postoperative spinopelvic alignment following long-segment fusion terminating at the sacrum. Given the retrospective study design and the unequal distribution of ALIF, these findings should be interpreted as hypothesis-generating rather than causal but may support consideration of spinopelvic fixation in patients at increased risk of distal construct failure.

## Introduction

Long-segment fusion to the sacrum represents one of the most demanding procedures in adult spine surgery and is routinely performed for adult spinal deformity, degenerative scoliosis, fixed sagittal imbalance, and advanced multilevel degenerative lumbar disease requiring extensive stabilization. Although modern instrumentation techniques have substantially improved surgical correction and construct durability, the lumbosacral junction remains the biomechanically weakest region of long posterior constructs because of high bending moments and shear forces acting across the L5–S1 segment. These forces contribute to an increased risk of distal mechanical complications, including screw loosening, pseudarthrosis, rod fracture, and subsequent revision surgery [1–3].

The optimal distal fixation strategy remains an important topic in complex reconstructive spine surgery. Constructs terminating at S1 rely primarily on sacral pedicle screw fixation, whereas spinopelvic fixation extends the construct into the pelvis using either traditional iliac screws or S2-alar-iliac (S2AI) screws. Numerous biomechanical investigations have demonstrated that spinopelvic fixation increases construct stiffness, reduces motion at the lumbosacral junction, and decreases mechanical stress acting on S1 screws, thereby potentially reducing the risk of distal construct failure [2–5].

In contemporary spinal deformity surgery, spinopelvic fixation has become widely accepted for patients at increased risk of mechanical failure. Both iliac screw fixation and S2AI fixation provide enhanced distal anchorage, although they differ regarding implant prominence, soft tissue irritation, wound complications, and revision profiles [6–11]. Current evidence suggests that S2AI fixation provides biomechanical stability comparable to traditional iliac screws while offering advantages related to implant profile and wound morbidity [6, 8, 10].

Mechanical failure at the lumbosacral junction, however, is influenced by multiple patient- and surgery-related factors beyond distal fixation alone. Bone quality, construct length, sagittal correction, osteoporosis, body mass index, and anterior column support all contribute to construct durability. In particular, anterior lumbar interbody fusion (ALIF) at L5/S1 has consistently been associated with improved fusion rates and reduced pseudarthrosis by restoring anterior column support and promoting a more favorable biological environment for fusion [12, 13]. Consequently, distal fixation strategy should be interpreted within the broader context of overall construct design rather than as an isolated determinant of surgical success.

Although the biomechanical rationale for spinopelvic fixation is well established, comparatively few clinical studies have simultaneously evaluated mechanical complications, maintenance of sagittal alignment, revision burden, and longitudinal patient-reported outcomes within a single consecutive cohort using standardized follow-up. Furthermore, many previous investigations have focused primarily on implant survival or pseudarthrosis without integrating radiographic and functional recovery over time.

Therefore, the objective of the present retrospective cohort study was to compare sacral-only fixation and spinopelvic fixation in patients undergoing long-segment fusion terminating at the sacrum. We specifically evaluated mechanical complications, radiographic alignment, revision surgery, and functional recovery during a standardized two-year follow-up period. Given the retrospective design and the presence of multiple potential confounding factors, the observed associations are intended to improve clinical risk stratification and should be interpreted as hypothesis-generating rather than causal.

## Methods

### Study design and ethics

This retrospective comparative cohort study was conducted at the Department of Orthopaedic Surgery, Otto-von-Guericke University Magdeburg, Germany. Institutional review board approval was obtained prior to data collection (Ethics Committee No. 16/22). The requirement for informed consent was waived because of the retrospective design and complete pseudonymization of all patient data before analysis.

The study was designed and reported according to the Strengthening the Reporting of Observational Studies in Epidemiology (STROBE) guidelines.

### Patient population

A total of 100 consecutive adult patients (≥ 18 years; 50 patients per group) who underwent posterior instrumented long-segment lumbar fusion involving four or more motion segments terminating at the sacrum between January 2000 and December 2024 were included.

Patients were allocated according to distal fixation strategy into:

1. Sacral-only fixation, consisting of bilateral S1 pedicle screw fixation without pelvic instrumentation.
2. Spinopelvic fixation, consisting of bilateral S1 fixation supplemented by pelvic fixation using either traditional iliac screws or S2-alar-iliac (S2AI) screws.

The primary objective of the study was to compare distal fixation strategies rather than individual pelvic fixation techniques. Therefore, iliac screws and S2AI screws were analyzed as one spinopelvic fixation cohort. Technique-specific subgroup analyses were considered inappropriate because of insufficient sample size.

Primary surgical indications included:

- adult spinal deformity,
- degenerative lumbar scoliosis,
- fixed sagittal imbalance,
- and advanced multilevel degenerative lumbar disease requiring long-segment stabilization.

Patients undergoing surgery for trauma, spinal infection, neoplastic disease, or neuromuscular scoliosis were excluded.

Because of the retrospective study design, no matching procedure was performed. The choice of distal fixation strategy reflected routine clinical decision-making and depended on several factors including construct length, perceived mechanical demands, sagittal alignment, bone quality, anatomical considerations, surgeon preference, and the surgical standards applicable during the respective treatment period.

### Surgical technique

All procedures were performed at a tertiary academic spine center by experienced spine surgeons.

Sacral-only constructs terminated at S1 using bilateral pedicle screws.

Spinopelvic constructs were extended to the pelvis using either iliac screws or S2-alar-iliac screws according to surgeon preference and individual anatomical considerations.

Anterior lumbar interbody fusion (ALIF) at L5/S1 was performed selectively to improve anterior column support in patients with severe disc degeneration, sagittal imbalance, poor bone quality, or increased mechanical demands. Because ALIF was more frequently performed in the spinopelvic fixation cohort, it was considered an important potential confounding factor during interpretation of fusion-related outcomes.

### Radiological assessment

Standing full-length anteroposterior and lateral radiographs were obtained preoperatively and postoperatively at:

- 6 weeks,
- 3 months,
- 6 months,
- 1 year,
- and 2 years.

Radiological measurements were performed using standardized adult spinal deformity measurement protocols based on previously established radiographic definitions [14–16].

The following parameters were assessed:

- sagittal vertical axis (SVA),
- pelvic incidence–lumbar lordosis (PI–LL) mismatch,
- pelvic tilt (PT),
- sacral slope (SS),
- and L4–S1 lordosis.

Because standardized full-length radiographs consistently visualizing the upper thoracic spine were not available throughout the entire study period, global alignment parameters such as T1 pelvic angle (T1PA) could not be reliably assessed across all patients and were therefore not included.

Radiographic assessment was performed by one investigator experienced in adult spinal deformity measurements using predefined measurement protocols. Formal intraobserver and interobserver reliability testing was not performed.

### Mechanical complications

Implant-related complications were systematically assessed, including:

- screw loosening,
- pseudarthrosis,
- rod fracture,
- and revision surgery.

Clinically relevant screw loosening was defined as a radiolucent zone of ≥ 1 mm surrounding the implant on standing radiographs according to previously validated radiographic criteria [17, 18].

The analysis evaluated the patient-level occurrence of clinically relevant screw loosening rather than the number or severity of loosened screws.

Grading systems describing the severity of screw loosening (e.g., Shimizu classification) were not applied because historical imaging over the entire 24-year inclusion period did not permit reliable retrospective grading.

Computed tomography (CT) was not routinely performed in all patients. CT imaging was obtained selectively in symptomatic patients or when conventional radiographs were inconclusive regarding implant integrity or fusion status. Consequently, radiographic assessment primarily relied on conventional standing radiographs, supplemented by CT when clinically indicated.

Pseudarthrosis at L5/S1 was defined by one or more of the following criteria:

- absence of continuous osseous bridging across the fusion segment,
- radiographic implant failure,
- progressive implant loosening,
- persistent mechanical pain requiring revision,

Consistent with previously published definitions [19, 20].

Revision procedures were individually reviewed and categorized according to their primary indication, including mechanical failure, pseudarthrosis, symptomatic screw loosening, wound complications, implant-related discomfort, infection, or adjacent segment pathology.

### Clinical outcomes

Clinical outcomes were assessed using the Oswestry Disability Index, a validated patient-reported outcome measure for spinal disorders [21, 22].

ODI questionnaires were collected:

- preoperatively,
- and at all postoperative follow-up intervals.

Scores were calculated as percentages, with higher values indicating greater disability.

Patients with incomplete ODI data at a given follow-up interval were excluded from the respective time-point analysis in accordance with accepted recommendations for handling missing PROM data in retrospective cohort studies [23].

Postoperative complications, including wound-related complications and revision procedures, were extracted from electronic medical records.

### Statistical analysis

Statistical analyses were performed using IBM SPSS Statistics Version 25.0 (IBM Corp., Armonk, NY, USA).

An a priori sample size calculation was performed for the primary endpoint of pseudarthrosis at L5/S1. Assuming pseudarthrosis rates of 40% after sacral-only fixation and 15% after spinopelvic fixation, a two-sided comparison of independent proportions (α = 0.05; power = 80%) yielded a required sample size of 49 patients per treatment group. To compensate for incomplete follow-up, 50 patients were included in each cohort.

Continuous variables were assessed for normality using the Shapiro–Wilk test.

Normally distributed continuous variables were compared using independent-samples t-tests, whereas non-normally distributed variables were analyzed using the Mann–Whitney U test.

Categorical variables were compared using Pearson’s chi-square test or Fisher’s exact test where appropriate.

Longitudinal radiographic and clinical outcomes were summarized descriptively across predefined follow-up intervals. Complication rates represent cumulative incidences up to the respective follow-up unless otherwise specified.

Unadjusted odds ratios (ORs) with corresponding 95% confidence intervals (CIs) were calculated to quantify associations between distal fixation strategy and major mechanical complications.

A multivariable regression model was deliberately not performed. Although adjustment for a limited number of variables would have been technically feasible, we considered such analyses potentially misleading because several clinically relevant confounding variables were incompletely available over the 24-year study period. These included bone mineral density, osteoporosis status, Hounsfield unit measurements, implant type, construct complexity, surgical era, surgeon preference, and the selective use of anterior lumbar interbody fusion (ALIF).

Adjustment for only a subset of these variables could have introduced residual confounding while implying a level of causal inference that cannot be justified in a retrospective observational study. Consequently, the reported associations should be interpreted as descriptive and hypothesis-generating rather than causal.

Statistical significance was defined as a two-sided *p* value < 0.05.

## Results

### Patient characteristics

A total of 100 consecutive patients met the inclusion criteria and completed the minimum clinical follow-up required for analysis. Fifty patients underwent sacral-only fixation, whereas fifty patients received spinopelvic fixation extending to the pelvis.

Baseline demographic and surgical characteristics are summarized in Table 1.

**Table 1:** Patient demographics and baseline characteristics. Values are presented as mean ± standard deviation (SD) or number (%). *P*-values represent between-group comparisons

Variable	Sacral-only fixation (*n* = 50)	Spinopelvic fixation (*n* = 50)	*p*-value
Age (years)	68.5 ± 8.5	67.6 ± 7.6	0.58
Female sex	27 (54%)	31 (62%)	0.41
BMI (kg/m²)	28.8 ± 3.7	28.3 ± 4.9	0.56
ASA class	2.9 ± 0.7	2.8 ± 0.7	0.49
Smoking	14 (28%)	14 (28%)	1.00
Diabetes mellitus	10 (20%)	14 (28%)	0.34
Instrumented levels	8.5 ± 1.7	8.5 ± 1.4	0.94
ALIF L5–S1	7 (14%)	23 (46%)	< 0.001

The two cohorts were generally comparable with respect to age (68.5 ± 8.5 vs. 67.6 ± 7.6 years), body mass index (28.8 ± 3.7 vs. 28.3 ± 4.9 kg/m²), American Society of Anesthesiologists (ASA) classification, smoking status, diabetes prevalence, and construct length.

The mean number of instrumented vertebral levels was identical between groups (8.5 ± 1.7 vs. 8.5 ± 1.4).

Anterior lumbar interbody fusion (ALIF) at L5/S1 was performed significantly more frequently in the spinopelvic fixation cohort than in the sacral-only cohort (46% vs. 14%, *p* < 0.01).

No other clinically relevant baseline differences were observed.

### Radiographic outcomes

Radiographic outcomes are summarized in Tables 2, 3, 4 and 5. 

**Table 2:** Sagittal vertical axis (SVA) measurements at baseline and during postoperative follow-up. Values are presented as mean ± SD in millimetres

Follow-up	Sacral-only fixation	Spinopelvic fixation	Between-group *p*-value
Baseline	75.1 ± 22.0	73.8 ± 23.1	0.77
6 weeks	37.5 ± 17.8	28.8 ± 18.3	0.03
3 months	35.6 ± 19.0	31.4 ± 19.4	0.28
6 months	36.6 ± 16.8	29.2 ± 16.3	0.04
1 year	26.0 ± 22.8	26.7 ± 17.9	0.87
2 years	29.0 ± 14.1	29.0 ± 21.7	1.00

**Table 3:** Pelvic incidence–lumbar lordosis (PI–LL) mismatch measured at baseline and during postoperative follow-up. Values are presented as mean ± SD in degrees. Lower values indicate improved sagittal alignment

Follow-up	Sacral-only fixation	Spinopelvic fixation	Between-group *p*-value
Baseline	20.8 ± 10.0	20.4 ± 8.6	0.81
6 weeks	9.6 ± 6.5	8.5 ± 6.6	0.42
3 months	11.4 ± 7.6	6.9 ± 8.1	0.01
6 months	10.8 ± 7.5	9.3 ± 6.0	0.29
1 year	9.8 ± 6.3	7.7 ± 7.2	0.13
2 years	8.4 ± 6.8	5.6 ± 7.7	0.04

**Table 4:** Mechanical complications during the two-year follow-up. Values are presented as number (%). Rates represent cumulative incidences during the two-year follow-up

Outcome	Sacral-only fixation	Spinopelvic fixation	OR (95% CI)	*p*-value
Screw loosening	17 (34%)	3 (6%)	3.43 (1.46–8.06)	0.004
Pseudarthrosis	24 (48%)	6 (12%)	8.43 (1.79–39.70)	< 0.001
Rod fracture	1 (2%)	1 (2%)	Not calculated	Not calculated
Revision surgery	11 (22%)	13 (26%)	0.80 (0.32–2.02)	0.66
Wound complication	8 (16%)	5 (10%)	1.71 (0.52–5.66)	0.38

Odds ratios (ORs) represent unadjusted patient-level associations with corresponding 95% confidence intervals (CIs). Because only one rod fracture occurred in each group, comparative estimates were not calculated for this outcome. Minor variations in the number of radiographically evaluable patients at individual follow-up visits resulted from revision surgery with implant exchange and incomplete radiographic follow-up

**Table 5:** Indications for revision surgery. Values represent clinically relevant indications leading to revision surgery

Indication	Sacral-only fixation	Spinopelvic fixation
Screw loosening	5	1
Pseudarthrosis	4	1
Rod fracture	1	1
Wound complication	0	5
Implant prominence / pain	0	3
Infection	1	1
Adjacent segment pathology	0	1
Other	0	1

One patient in the spinopelvic fixation cohort underwent revision for two clinically relevant indications and is therefore represented in both categories. Early revisions in the spinopelvic fixation cohort were predominantly related to wound complications or implant-related symptoms, whereas revisions following sacral-only fixation were mainly associated with mechanical failure

#### Screw loosening

The incidence of clinically relevant screw loosening increased progressively during follow-up in both treatment groups but occurred substantially more frequently following sacral-only fixation.

At two years, screw loosening was identified in 17 of 50 patients (34.0%) in the sacral-only fixation group compared with 3 of 50 patients (6.0%) in the spinopelvic fixation group (*p* = 0.004, OR 3.43, 95% CI 1.46–8.06).

The apparent reduction in screw loosening between the one-year and two-year follow-up within the sacral-only cohort reflects patients who underwent revision surgery with implant exchange before the final follow-up as well as incomplete radiographic follow-up in a small number of cases. Accordingly, cumulative complication rates should be interpreted together with the number of evaluable patients at each follow-up interval.

**Fig. 1:**
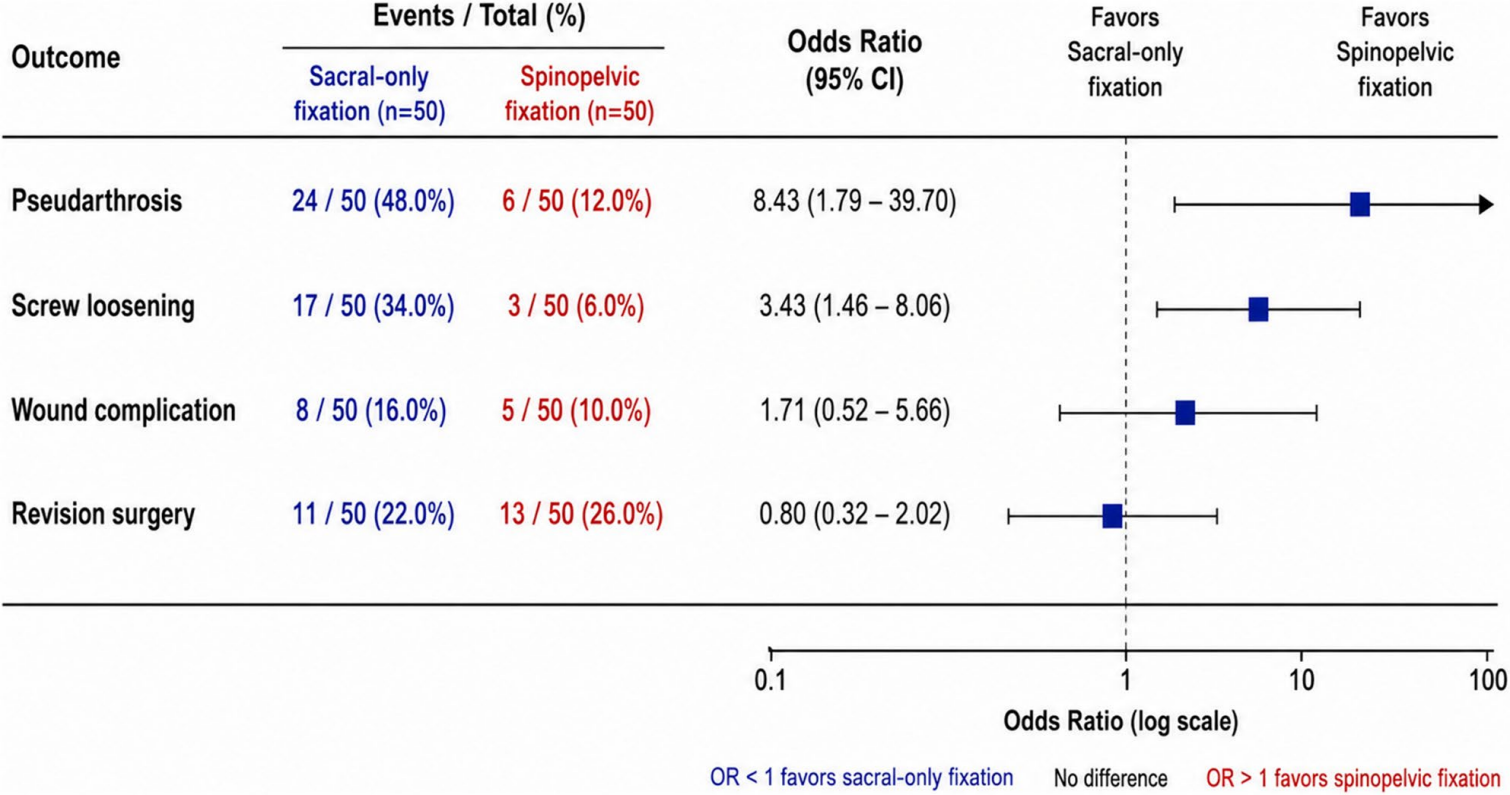
Forest plot of unadjusted odds ratios for major mechanical outcomes at two years. Forest plot illustrating unadjusted odds ratios (ORs) with 95% confidence intervals comparing sacral-only fixation and spinopelvic fixation for the principal study outcomes at the two-year follow-up. Values greater than 1 indicate a higher likelihood of the respective outcome following sacral-only fixation, whereas values less than 1 favor sacral-only fixation. Event numbers and cumulative incidences for both treatment groups are presented alongside each outcome. Odds ratios were calculated on a patient level

Overall mechanical complications are summarized in Table 4 and illustrated graphically in Fig. 1.

#### Pseudarthrosis

Pseudarthrosis at L5/S1 was observed considerably more frequently following sacral-only fixation.

At the two-year follow-up, pseudarthrosis was diagnosed in 24 of 50 patients (48.0%) after sacral-only fixation compared with 6 of 50 patients (12.0%) after spinopelvic fixation (*p* < 0.01), an unadjusted odds ratio of 8.43 (95% CI 1.79–39.70).

Given the higher utilization of ALIF in the spinopelvic fixation cohort, these findings should be interpreted with consideration of potential confounding by anterior column support.

**Fig. 2:**
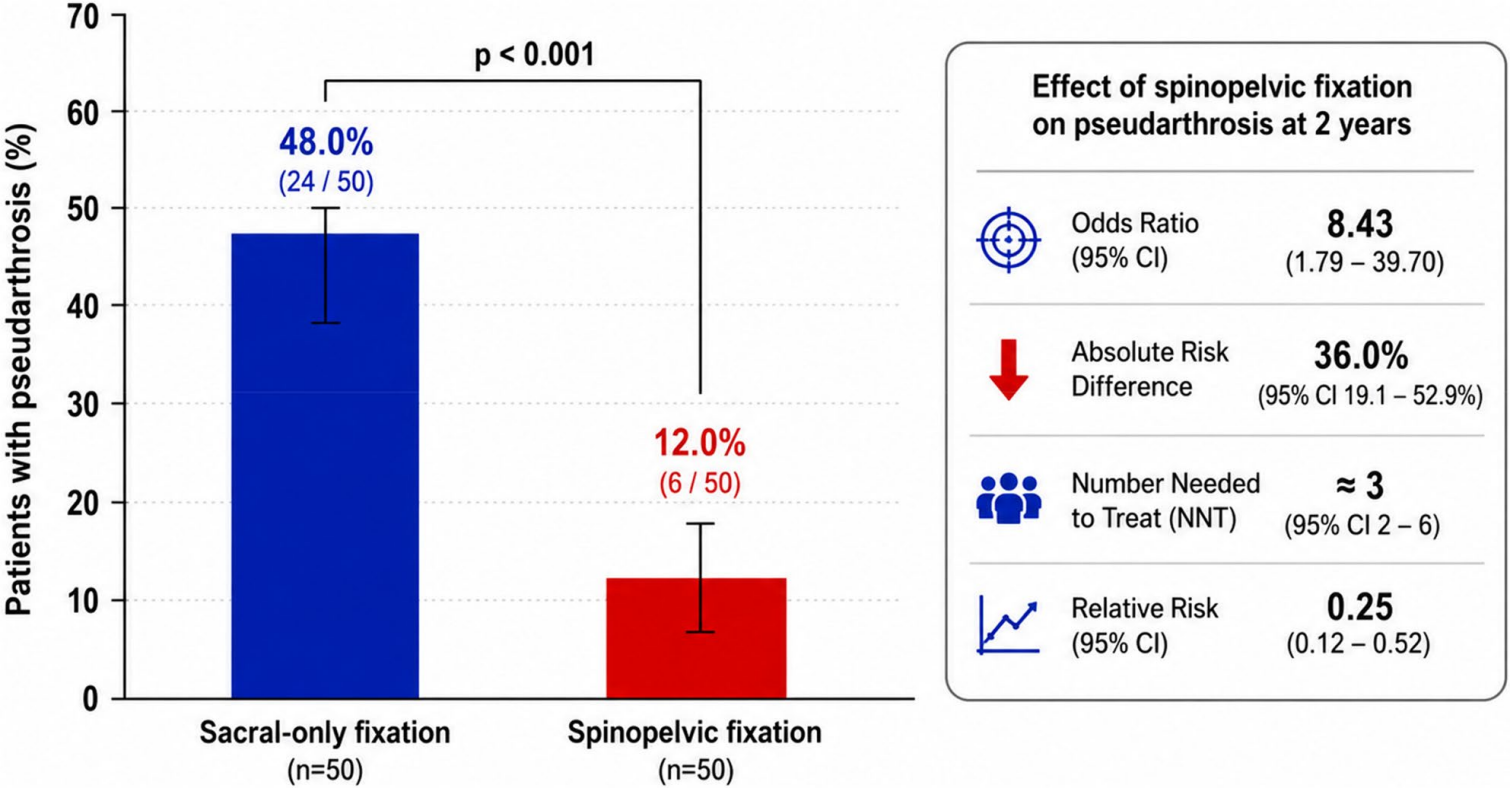
Incidence of pseudarthrosis at the two-year follow-up. Comparison of cumulative pseudarthrosis rates at L5–S1 two years after surgery in patients undergoing sacral-only fixation and spinopelvic fixation. Bars represent cumulative incidence with 95% confidence intervals. The accompanying summary panel presents the unadjusted odds ratio (OR), absolute risk difference, relative risk (RR), and estimated number needed to treat (NNT). Because of the retrospective observational design and unequal distribution of anterior lumbar interbody fusion (ALIF), these effect estimates represent clinical associations rather than causal effects

The cumulative incidence of pseudarthrosis at two years is additionally illustrated in Fig. 2.

#### Sagittal alignment

Both treatment groups demonstrated substantial postoperative improvement in sagittal alignment.

Sagittal vertical axis (SVA) improved markedly after surgery and remained stable throughout follow-up. Final SVA values were comparable between groups at two years (29.0 ± 14.1 mm vs. 29.0 ± 21.7 mm).

**Fig. 3:**
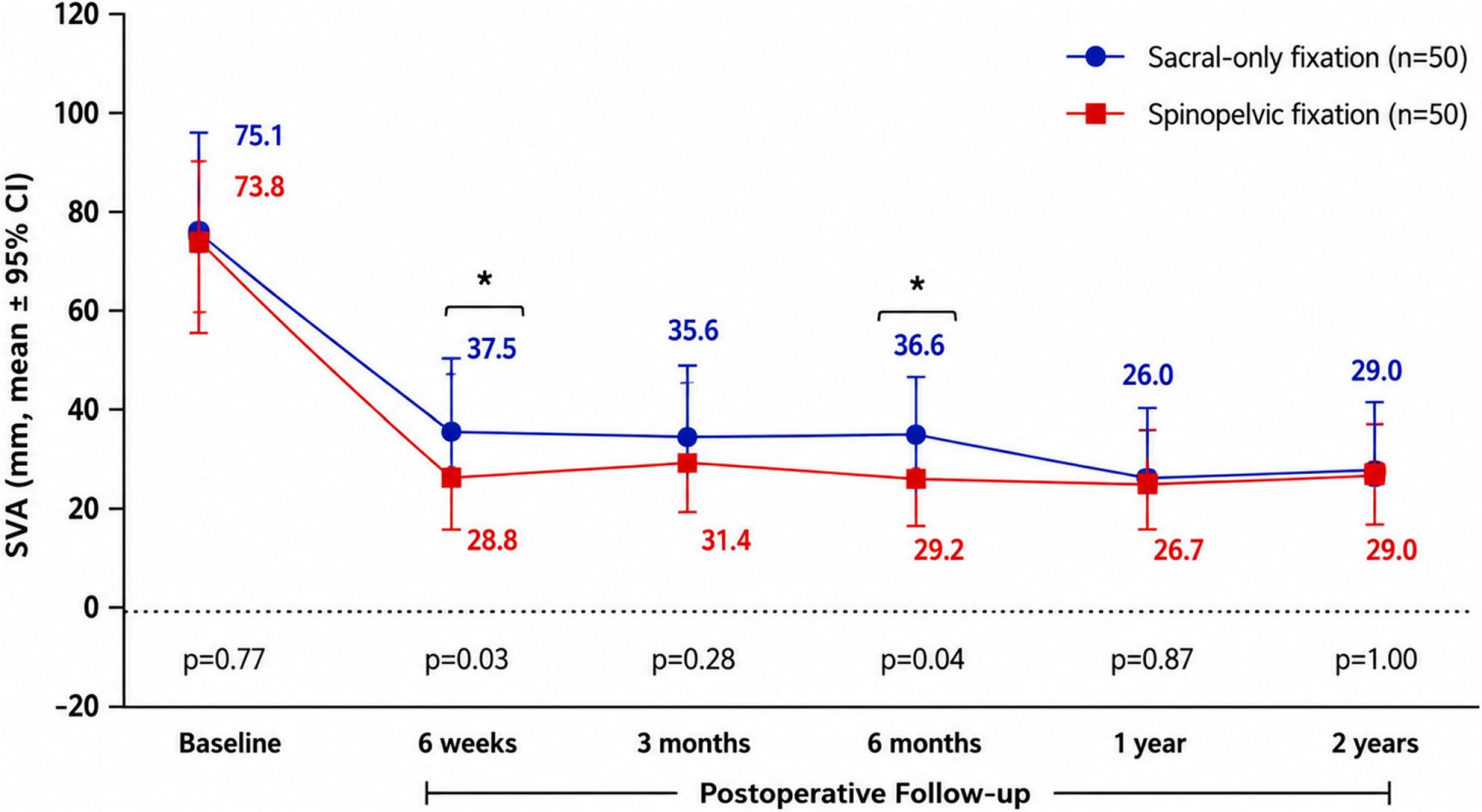
Sagittal vertical axis (SVA) during the two-year follow-up. Mean sagittal vertical axis (SVA) measurements are presented from baseline through the two-year follow-up for the sacral-only fixation (blue circles) and spinopelvic fixation (red squares) cohorts. Error bars represent 95% confidence intervals. Between-group *p*-values are shown below each follow-up interval. Positive SVA values indicate anterior sagittal imbalance. Significant between-group differences are indicated by an asterisk (*p* < 0.05)

Longitudinal changes in sagittal vertical axis are illustrated in Fig. 3.

Pelvic incidence–lumbar lordosis (PI–LL) mismatch improved postoperatively in both cohorts. However, patients undergoing spinopelvic fixation demonstrated consistently lower PI–LL mismatch throughout follow-up, with significantly lower values at the two-year examination (5.6° ± 7.7 vs. 8.4° ± 6.8; *p* < 0.05), indicating improved maintenance of sagittal correction.

**Fig. 4:**
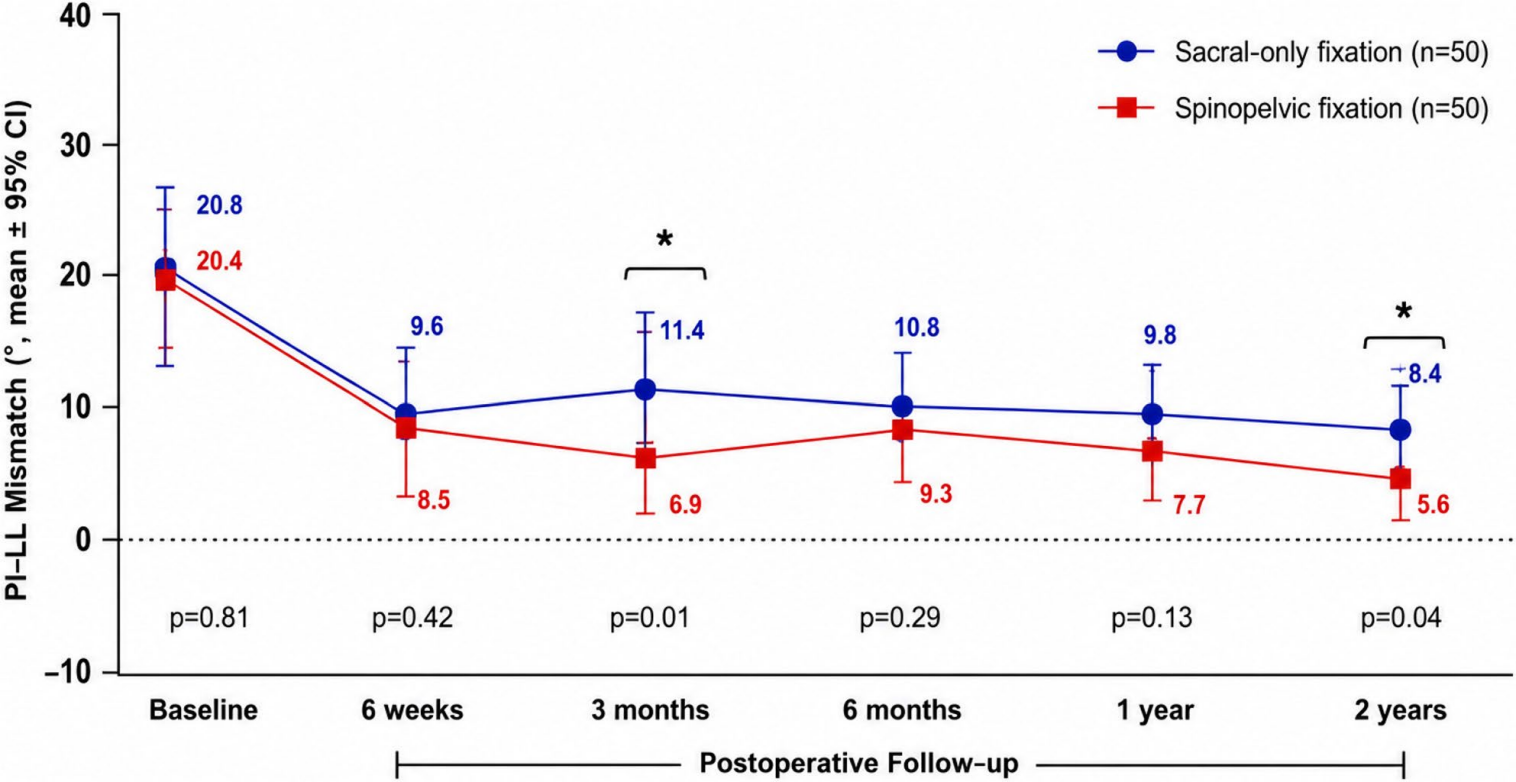
Pelvic incidence–lumbar lordosis (PI–LL) mismatch during the two-year follow-up. Mean pelvic incidence–lumbar lordosis (PI–LL) mismatch is shown preoperatively and throughout the postoperative follow-up for both fixation strategies. Error bars represent 95% confidence intervals. Lower PI–LL mismatch values indicate improved sagittal alignment. Between-group *p*-values are displayed for each follow-up interval. Significant differences are indicated by an asterisk (*p* < 0.05)

Longitudinal changes in PI–LL mismatch are illustrated in Fig. 4.

### Clinical outcomes

**Fig. 5:**
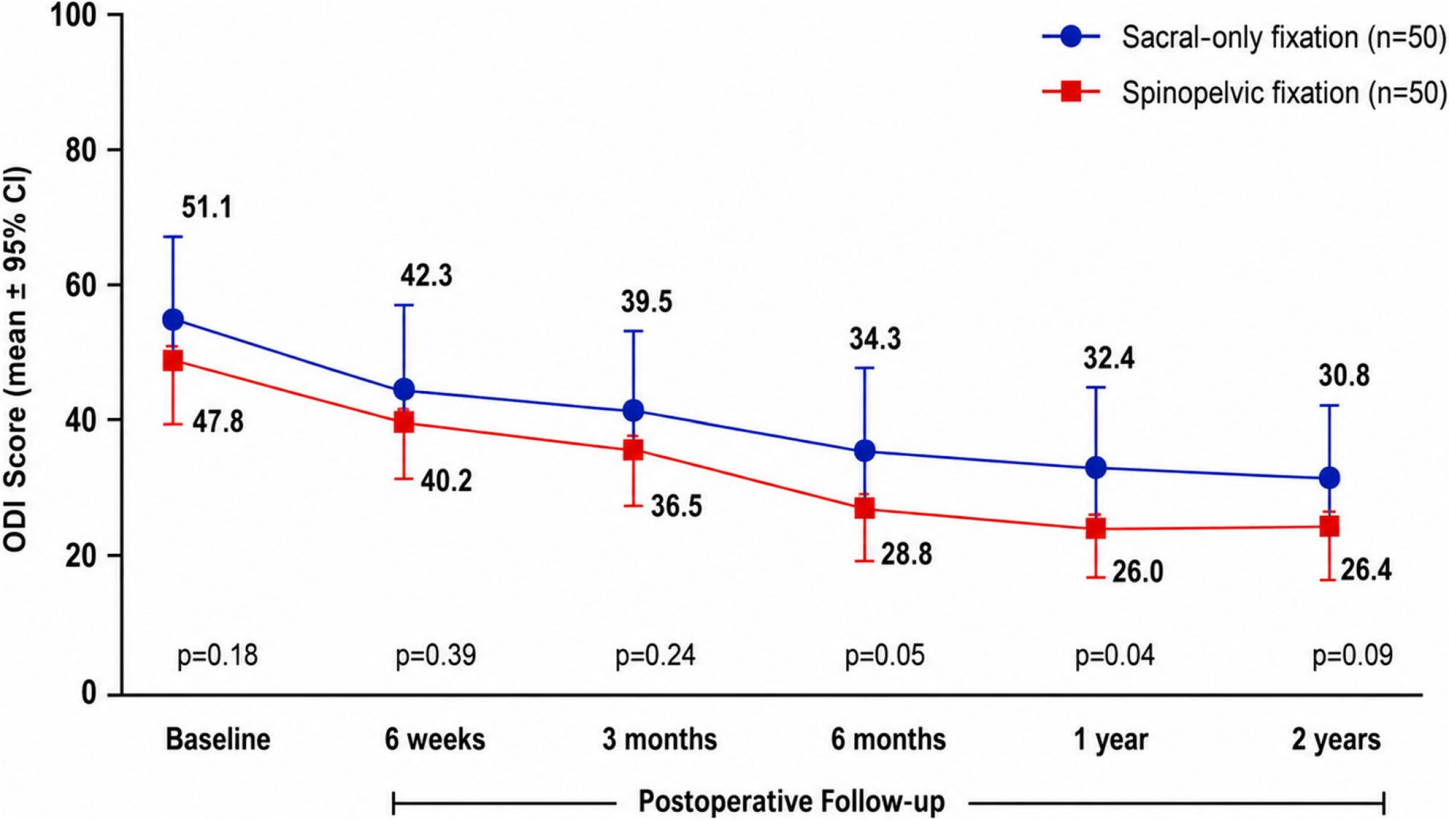
Longitudinal functional outcome measured by the Oswestry Disability Index (ODI). Mean Oswestry Disability Index (ODI) scores are shown preoperatively and at 6 weeks, 3 months, 6 months, 1 year, and 2 years after surgery for patients undergoing sacral-only fixation (blue circles) and spinopelvic fixation (red squares). Error bars represent 95% confidence intervals. Between-group *p*-values are shown for each follow-up interval. Lower ODI scores indicate less disability and better functional outcome

Clinical outcomes are summarized in Table 6; Fig. 5.

**Table 6:** Clinical outcomes assessed by Oswestry Disability Index (ODI) scores at baseline and during postoperative follow-up. Values are presented as mean ± SD. Lower scores indicate lower disability

Follow-up	Sacral-only fixation	Spinopelvic fixation	Between-group *p*-value
Baseline	51.1 ± 12.0	47.8 ± 12.0	0.18
6 weeks	42.3 ± 12.2	40.2 ± 12.9	0.39
3 months	39.5 ± 13.2	36.5 ± 12.1	0.24
6 months	34.3 ± 14.1	28.8 ± 12.8	0.05
1 year	32.4 ± 14.0	26.0 ± 15.2	0.04
2 years	30.8 ± 13.0	26.4 ± 12.7	0.09

Both treatment groups demonstrated continuous postoperative improvement in functional disability as measured by the Oswestry Disability Index (ODI).

Baseline ODI scores were comparable between groups (51.1 ± 12.0 vs. 47.8 ± 12.0).

Patients treated with spinopelvic fixation demonstrated numerically greater improvement during the early postoperative period, with lower ODI scores observed from six weeks through one year.

At the final two-year follow-up, mean ODI values remained slightly lower in the spinopelvic fixation group (26.4 ± 12.7) compared with the sacral-only fixation group (30.8 ± 13.0), although the absolute between-group difference was modest.

Overall, both fixation strategies resulted in clinically meaningful functional improvement throughout follow-up.

### Revision surgery

**Fig. 6:**
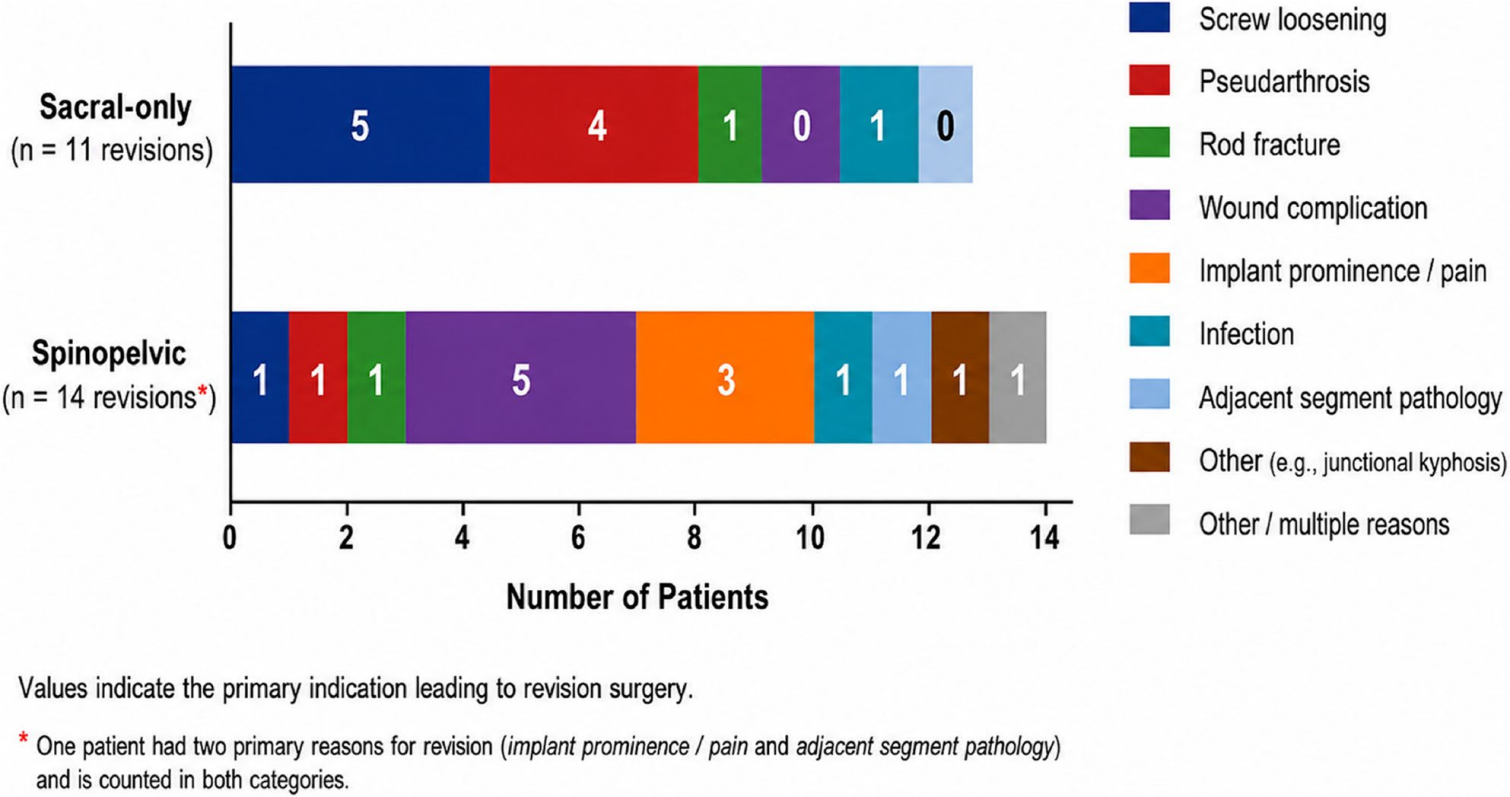
Primary indications for revision surgery. Distribution of the primary indication leading to revision surgery in patients undergoing sacral-only fixation and spinopelvic fixation. Horizontal stacked bars display the number of revision procedures attributed to each indication, including screw loosening, pseudarthrosis, rod fracture, wound complications, implant prominence or pain, infection, adjacent segment pathology, and other causes. In the spinopelvic fixation cohort, one patient underwent revision for two clinically relevant indications and is therefore represented in both categories. Overall revision frequency was comparable between groups; however, revisions following sacral-only fixation were predominantly associated with mechanical failure, whereas revisions after spinopelvic fixation more frequently reflected wound-related or implant-related complications

Overall revision rates are presented in Table 4, whereas the underlying indications for revision surgery are summarized separately in Table 5 and illustrated in Fig. 6.

Revision surgery was required in both treatment groups during the two-year observation period.

Overall revision rates were similar between groups (22.0% after sacral-only fixation versus 26.0% after spinopelvic fixation).

Within the sacral-only fixation cohort, revision procedures were predominantly performed because of symptomatic screw loosening, pseudarthrosis at L5/S1, rod-related mechanical failure, or progressive construct instability.

In contrast, revision procedures following spinopelvic fixation occurred primarily during the early postoperative period and were more frequently related to wound complications, implant prominence, or implant-related soft tissue irritation than to mechanical nonunion.

Consequently, comparable overall revision frequencies should not be interpreted as reflecting equivalent mechanisms of failure.

### Postoperative complications

Early wound complications occurred infrequently in both treatment groups.

Most wound-related complications developed within the first three postoperative months and were managed successfully without long-term sequelae.

No statistically significant differences were observed regarding overall wound complication rates.

Rod fracture occurred infrequently and was primarily associated with cases of pseudarthrosis requiring subsequent revision surgery.

### Summary of major outcomes

Compared with sacral-only fixation, spinopelvic fixation was associated with:

- significantly lower rates of clinically relevant screw loosening,
- significantly lower pseudarthrosis rates,
- improved maintenance of PI–LL mismatch,
- earlier postoperative functional recovery,
- but similar overall revision frequencies, with markedly different underlying indications for reoperation.

Because of the retrospective study design and the unequal distribution of anterior lumbar interbody fusion (ALIF), these findings represent clinical associations rather than evidence of causality.

## Discussion

### Principal findings

The present retrospective cohort study compared sacral-only fixation with spinopelvic fixation in patients undergoing long-segment fusion terminating at the sacrum. Four principal findings emerged from this analysis. First, spinopelvic fixation was associated with significantly lower rates of clinically relevant screw loosening and pseudarthrosis during the two-year follow-up. Second, patients undergoing spinopelvic fixation were associated with improved maintenance of postoperative sagittal alignment, as reflected by a lower pelvic incidence–lumbar lordosis (PI–LL) mismatch at final follow-up, whereas sagittal vertical axis (SVA) remained comparable between groups. Third, although overall revision rates were similar, the underlying indications for revision differed substantially between fixation strategies, with predominantly mechanical failures following sacral-only fixation and mainly wound- or implant-related complications after spinopelvic fixation. Finally, both treatment strategies resulted in clinically meaningful improvement in patient-reported disability, although functional recovery occurred earlier in the spinopelvic fixation group.

Collectively, these findings are consistent with the concept that extending long posterior constructs to the pelvis may improve distal construct durability and maintenance of lumbosacral stability. However, given the retrospective observational design and the presence of multiple potential confounding factors, these associations should be interpreted cautiously and regarded as hypothesis-generating rather than evidence of a causal relationship.

### Mechanical complications and construct stability

Mechanical failure at the lumbosacral junction remains one of the most challenging complications following long-segment spinal fusion. The transition between the mobile lumbar spine and the rigid pelvis exposes the distal construct to considerable flexion moments, axial loading, and shear forces, making S1 pedicle screws particularly vulnerable to loosening and nonunion. Previous biomechanical investigations have consistently demonstrated that extending fixation into the pelvis distributes these forces more effectively, reduces motion at the lumbosacral junction, and increases construct stiffness compared with sacral-only fixation [4, 5].

Our findings are consistent with these biomechanical principles. Patients treated with sacral-only fixation demonstrated substantially higher rates of clinically relevant screw loosening and pseudarthrosis than those receiving spinopelvic fixation. Similar observations have previously been reported by Kuklo et al. and Tsuchiya et al., who demonstrated superior lumbosacral fusion rates and lower mechanical failure rates following sacropelvic fixation in adult spinal deformity surgery [2, 3]. More recently, Odland et al. identified screw loosening as the predominant mechanical failure mode in long constructs terminating at S1 and highlighted the importance of optimizing distal fixation to reduce construct-related complications [1]. The present study extends these observations by demonstrating comparable findings within a contemporary consecutive clinical cohort while simultaneously evaluating radiographic alignment and functional recovery.

Importantly, the purpose of the present study was not to re-establish the biomechanical superiority of pelvic fixation, which has already been convincingly demonstrated in experimental and finite-element studies [4, 5]. Rather, our data provide additional clinical evidence by integrating mechanical complications, radiographic alignment, revision surgery, and patient-reported outcomes within a single standardized two-year follow-up. This comprehensive evaluation represents an important addition to the existing literature, in which these outcome domains have often been investigated separately.

Although spinopelvic fixation demonstrated superior mechanical performance, the occurrence of radiographic failure should not automatically be equated with clinical failure. Several patients with radiographic screw loosening or pseudarthrosis remained clinically compensated and were therefore managed non-operatively. Revision surgery was reserved for patients with symptomatic mechanical failure, progressive instability, persistent pain, or implant-related complications. This distinction underscores the importance of interpreting radiographic outcomes within the broader clinical context rather than considering imaging findings in isolation.

### Sagittal alignment

Restoration and maintenance of sagittal alignment have become fundamental objectives of modern spinal deformity surgery because postoperative spinopelvic balance is closely associated with functional recovery, mechanical complications, and long-term health-related quality of life [14–16]. In the present study, both fixation strategies achieved substantial correction of sagittal deformity immediately after surgery, indicating that adequate surgical correction was obtained irrespective of distal fixation technique.

Interestingly, despite comparable final SVA values, patients undergoing spinopelvic fixation demonstrated significantly lower PI–LL mismatch throughout follow-up and maintained this advantage at two years. This finding suggests that improved distal construct stability may contribute to preservation of lumbar lordosis during postoperative remodeling rather than influencing global sagittal balance directly. Similar observations have previously been reported by Oba et al., who demonstrated gradual loss of lumbar lordosis following long-segment fusion despite initially satisfactory deformity correction, emphasizing the importance of durable distal fixation for maintaining sagittal alignment [24].

The absence of significant differences in SVA at final follow-up is not unexpected. Global sagittal balance reflects a complex interaction between spinal alignment, pelvic compensation, lower-extremity mechanics, and individual patient morphology rather than distal fixation alone [14–16]. Consequently, preservation of PI–LL mismatch may represent a more sensitive indicator of distal construct integrity than SVA in the present cohort.

Although the absolute differences in PI–LL mismatch were modest, even relatively small losses of sagittal correction have been associated with increased rates of mechanical complications, adjacent segment degeneration, and inferior patient-reported outcomes after adult spinal deformity surgery [14, 19]. From a clinical perspective, maintaining postoperative spinopelvic alignment may therefore represent an additional advantage of spinopelvic fixation beyond simple reduction of implant-related complications.

It should also be acknowledged that contemporary global alignment parameters such as the T1 pelvic angle (T1PA) were not evaluated in the present study because standardized full-length standing radiographs consistently including the upper thoracic spine were not available throughout the entire inclusion period. Restricting the analysis to SVA and PI–LL ensured methodological consistency across the complete cohort while allowing comparison of radiographic parameters available in all patients.

### Functional outcome

Both treatment strategies resulted in substantial postoperative improvement in disability as measured by the Oswestry Disability Index, confirming the effectiveness of long-segment reconstruction in appropriately selected patients. The observed improvement is consistent with previous studies demonstrating meaningful functional recovery following correction of adult spinal deformity and advanced degenerative lumbar disease [21, 22].

Although final ODI scores were relatively similar between groups, patients treated with spinopelvic fixation demonstrated numerically greater improvement during the early postoperative period and maintained lower disability scores throughout most follow-up intervals. While these differences did not translate into marked long-term separation of ODI values, they may indicate that improved distal construct stability may facilitate earlier rehabilitation and more rapid functional recovery.

At the same time, the relatively small difference in final ODI scores highlights that patient-reported outcome is influenced by substantially more than mechanical construct integrity alone. Recovery following complex spinal reconstruction depends on numerous interacting factors, including neurological status, muscular function, rehabilitation, pain perception, psychosocial characteristics, medical comorbidities, and patient expectations [14, 21, 22]. Consequently, mechanical success does not necessarily translate into proportionally greater improvement in patient-reported disability.

This observation further emphasizes the importance of evaluating both radiographic and functional outcomes when assessing distal fixation strategies. Whereas spinopelvic fixation appears to reduce mechanical complications and better preserve sagittal alignment, the ultimate clinical benefit should be interpreted within the broader context of patient-centered recovery and individualized treatment goals.

### Interpretation of pseudarthrosis and the potential influence of ALIF

One of the most striking findings of the present study was the substantially lower rate of pseudarthrosis observed in the spinopelvic fixation cohort. However, this result warrants careful interpretation because fusion success following long-segment reconstruction is influenced by numerous biological and mechanical factors beyond the distal fixation strategy itself.

Most importantly, anterior lumbar interbody fusion (ALIF) at L5–S1 was performed considerably more frequently in the spinopelvic fixation group than in the sacral-only cohort. This imbalance represents an important potential source of confounding. Previous clinical studies have consistently demonstrated that ALIF provides superior anterior column support, restores disc height, increases the fusion surface area, may improve load sharing across the lumbosacral junction, and is associated with lower rates of pseudarthrosis compared with posterior interbody techniques [12, 13]. Consequently, the lower incidence of pseudarthrosis observed in the present cohort cannot reasonably be attributed solely to the distal fixation strategy.

Rather, our findings suggest that successful lumbosacral fusion is likely determined by the combined effect of multiple biomechanical and biological factors, including distal construct stability, anterior column support, bone quality, construct length, and patient-specific healing capacity. Spinopelvic fixation and ALIF should therefore not be regarded as competing strategies but as complementary components of a comprehensive reconstructive approach in patients at increased risk of mechanical failure.

The reviewer appropriately suggested adjustment for ALIF using multivariable regression analysis. Although logistic regression including a limited number of variables would have been statistically feasible, we deliberately chose not to present adjusted estimates. The principal limitation of the present dataset was not the number of observations but the incomplete availability of several clinically relevant confounders over the 24-year study period. Variables such as osteoporosis, bone mineral density, Hounsfield unit measurements, implant type, construct complexity, surgeon preference, and temporal evolution of surgical techniques were not consistently documented throughout the study period. Adjustment for ALIF alone or for only a subset of available variables would therefore have introduced substantial residual confounding while potentially implying a level of causal inference that cannot be justified in a retrospective observational study.

Accordingly, we considered a transparent presentation of unadjusted associations together with a comprehensive discussion of potential confounding to represent the more scientifically rigorous approach. The observed association should therefore be interpreted as multifactorial and hypothesis-generating rather than causal. Future prospective studies incorporating standardized anterior column reconstruction strategies and comprehensive multivariable adjustment are required to determine the independent contribution of pelvic fixation to fusion success.

### Revision surgery

Although the overall revision rate did not differ significantly between treatment groups, closer examination revealed marked differences in the underlying mechanisms leading to reoperation. This distinction represents an important clinical observation because revision surgery is frequently reported as a composite endpoint despite encompassing a broad spectrum of pathological processes.

Patients treated with sacral-only fixation underwent revision predominantly because of symptomatic mechanical failure, including progressive screw loosening, pseudarthrosis, rod fracture, or construct instability. These revisions generally occurred later during follow-up and reflected failure of the lumbosacral construct to maintain long-term mechanical integrity.

In contrast, revision procedures following spinopelvic fixation occurred primarily during the early postoperative period and were more commonly related to wound complications, implant prominence, soft tissue irritation, or other implant-related symptoms rather than biological failure of fusion. These findings are consistent with previous reports demonstrating that spinopelvic fixation, particularly with traditional iliac screws, may increase the risk of implant prominence and wound-related morbidity despite providing superior biomechanical stability [6, 8, 9].

Therefore, similar overall revision frequencies should not be interpreted as indicating equivalent construct performance. Rather, the underlying indication for revision provides important additional information regarding the mode of failure and the clinical consequences associated with each fixation strategy. Differentiating revision mechanisms may therefore be more informative than reporting overall revision rates alone.

### Pelvic fixation techniques: iliac screws versus S2-alar-iliac fixation

The present study compared sacral-only fixation with spinopelvic fixation as a treatment strategy rather than evaluating individual pelvic fixation techniques. Consequently, traditional iliac screws and S2-alar-iliac (S2AI) screws were analyzed together within the spinopelvic fixation cohort.

Current evidence suggests that both techniques provide comparable biomechanical stability while differing mainly with respect to implant profile, soft tissue irritation, wound complications, and ease of instrumentation [6–8, 10]. Several comparative studies have demonstrated lower implant prominence and fewer wound-related complications following S2AI fixation, whereas mechanical outcomes appear largely equivalent [6, 8, 10, 11].

Because the number of patients treated with each pelvic fixation technique was limited, statistically meaningful subgroup analyses were not considered appropriate. Consequently, our findings should be interpreted as reflecting the overall effect of spinopelvic fixation rather than the superiority of one pelvic fixation technique over another. Future multicenter studies with larger cohorts should directly compare contemporary iliac and S2AI fixation strategies using standardized radiographic and clinical outcome measures.

### Strengths of the study

The present study has several strengths. First, it represents a consecutive real-world cohort treated at a tertiary academic spine center with standardized clinical and radiographic follow-up over two years. Second, radiographic mechanical complications, sagittal alignment, patient-reported outcomes, and revision surgery were evaluated simultaneously, allowing a comprehensive assessment of distal fixation strategies. Third, unlike many previous investigations focusing exclusively on implant survival or pseudarthrosis, the present analysis integrates structural, functional, and clinical outcomes within a single study. Finally, detailed evaluation of revision indications provides additional insight into the different mechanisms of construct failure beyond overall revision frequency, thereby improving the clinical interpretation of the observed results.

### Overall interpretation

Taken together, the findings of the present study support the growing body of evidence indicating that spinopelvic fixation is associated with improved mechanical durability of long-segment constructs terminating at the sacrum. Patients undergoing spinopelvic fixation experienced lower rates of screw loosening and pseudarthrosis together with better preservation of postoperative spinopelvic alignment while achieving comparable long-term functional improvement.

However, these findings must be interpreted within the context of the retrospective study design and several important sources of residual confounding, including unequal use of ALIF, incomplete assessment of bone quality, changes in surgical techniques over the 24-year inclusion period, and surgeon-dependent treatment selection. Consequently, the observed associations cannot establish causality or isolate the independent effect of distal fixation strategy.

Instead, the present study provides clinically relevant observational evidence that may support preoperative risk stratification and individualized surgical planning in complex lumbosacral reconstruction. Prospective multicenter studies incorporating standardized assessment of bone quality, contemporary sagittal alignment parameters, uniform anterior column reconstruction, and adequately powered multivariable analyses are warranted to determine the independent contribution of distal fixation strategy to long-term construct survival and patient outcomes.

### Limitations

This study should be interpreted in light of several limitations inherent to its retrospective observational design.

First, the retrospective single-center design limits the ability to establish causal relationships and may reduce the generalizability of the findings to other institutions or surgical practices. Although all patients were treated at a high-volume academic spine center using standardized follow-up protocols, treatment allocation was not randomized and therefore remained subject to selection bias and surgeon-dependent decision-making.

Second, the unequal distribution of anterior lumbar interbody fusion (ALIF) at L5–S1 represents one of the most important potential confounding factors in the present study. ALIF was performed considerably more frequently in the spinopelvic fixation cohort and has previously been shown to improve anterior column support, reduce mechanical stress at the lumbosacral junction, and increase fusion rates in long-segment constructs [12, 13]. Consequently, the lower pseudarthrosis rate observed in the spinopelvic fixation group cannot be attributed solely to the distal fixation strategy. Instead, the observed association is likely multifactorial, reflecting the combined influence of anterior column support, construct stability, patient characteristics, and biological factors.

Third, although multivariable regression analysis was considered during study design, we deliberately refrained from presenting adjusted models. While regression including a limited number of variables would have been technically feasible, several clinically important confounders were incompletely available throughout the 24-year inclusion period. These included bone mineral density, osteoporosis status, Hounsfield unit measurements, implant type, construct complexity, surgical era, surgeon preference, and standardized assessment of anterior column reconstruction. Adjustment for only a subset of these variables would therefore have introduced substantial residual confounding while potentially implying a level of causal inference that cannot be justified in a retrospective cohort. We therefore considered transparent reporting of unadjusted associations together with comprehensive discussion of potential confounding to represent the more scientifically rigorous approach.

Fourth, bone quality could not be assessed systematically because dual-energy X-ray absorptiometry (DEXA), Hounsfield unit measurements, and standardized osteoporosis screening were not routinely available throughout the entire study period. Since reduced bone mineral density is a well-recognized risk factor for screw loosening, pseudarthrosis, and construct failure [1, 19, 25, 26], residual confounding by bone quality cannot be excluded.

Fifth, computed tomography (CT) was not routinely obtained in all patients but was performed selectively in symptomatic patients or when conventional radiographs were inconclusive. Although this approach reflects routine clinical practice, selective CT imaging may have introduced detection bias by influencing the reported rates of screw loosening and pseudarthrosis. Consequently, both underestimation and overestimation of radiographic failure remain possible.

Sixth, radiographic measurements were performed by one experienced investigator using standardized adult spinal deformity measurement protocols. Formal intraobserver and interobserver reliability analyses were not performed. Although standardized measurement techniques were applied consistently, some degree of measurement variability cannot be excluded.

Seventh, the study included patients treated over a prolonged period extending from 2000 to 2024. During this interval, substantial advances occurred in implant technology, navigation techniques, perioperative management, biological augmentation, and surgical strategies, including the increasing adoption of S2-alar-iliac fixation. These temporal changes may have influenced patient selection, operative decision-making, and clinical outcomes independently of the distal fixation strategy itself. Although subgroup analyses by surgical era were considered, such analyses would have substantially reduced statistical power and remained susceptible to multiple sources of residual confounding.

Finally, the present study evaluated spinopelvic fixation as a treatment strategy and therefore analyzed traditional iliac screws and S2-alar-iliac screws as one combined cohort. Because of the limited number of patients within each subgroup, meaningful comparisons between these two pelvic fixation techniques were not possible. Consequently, the present findings should be interpreted as reflecting the overall effect of spinopelvic fixation rather than the superiority of any individual pelvic fixation technique.

Despite these limitations, the study also possesses several important strengths. It represents a consecutive real-world cohort with standardized clinical and radiographic follow-up, comprehensive evaluation of mechanical, radiographic, functional, and revision-related outcomes, and clinically relevant long-term observations reflecting contemporary spinal reconstructive practice. The consistency of the observed associations across multiple outcome domains may support the clinical relevance of the findings while emphasizing that they should be interpreted as hypothesis-generating rather than causal.

### Clinical implications

The choice of distal fixation remains one of the most important decisions during planning of long-segment lumbosacral reconstruction. The present findings suggest that extending fixation into the pelvis may reduce the risk of distal mechanical complications while improving maintenance of postoperative spinopelvic alignment. These observations are particularly relevant in patients with recognized risk factors for construct failure, including extensive fusion length, osteoporosis or impaired bone quality, revision surgery, obesity, severe sagittal deformity, and increased mechanical demands at the lumbosacral junction.

At the same time, our findings emphasize that successful long-segment reconstruction cannot be attributed to the distal fixation strategy alone. Construct durability results from the interaction of multiple mechanical and biological factors, including restoration of sagittal alignment, optimization of anterior column support, implant selection, fusion biology, and patient-specific risk factors. Consequently, spinopelvic fixation should be considered one component of a comprehensive reconstructive strategy rather than an isolated intervention.

These findings may contribute to improved preoperative risk stratification and support individualized decision-making when selecting the optimal distal fixation strategy in complex spinal reconstruction.

## Conclusion

In this retrospective cohort study, spinopelvic fixation was associated with lower rates of clinically relevant screw loosening, reduced pseudarthrosis, and improved maintenance of postoperative spinopelvic alignment compared with sacral-only fixation following long-segment fusion terminating at the sacrum. Although overall revision rates were comparable, the underlying indications for revision differed substantially, with predominantly mechanical failure following sacral-only fixation and mainly wound- or implant-related complications after spinopelvic fixation. Both treatment strategies resulted in meaningful improvement in patient-reported functional outcome.

Because of the retrospective design and the presence of important potential confounding factors, including unequal utilization of anterior lumbar interbody fusion, incomplete assessment of bone quality, and temporal changes in surgical practice, the independent contribution of distal fixation strategy cannot be established. The observed associations should therefore be interpreted as multifactorial and hypothesis-generating rather than causal.

Nevertheless, the consistency of the observed radiographic and clinical findings suggests that spinopelvic fixation may represent an important component of contemporary long-segment lumbosacral reconstruction in appropriately selected patients. Prospective multicenter studies with standardized assessment of bone quality, anterior column support, contemporary sagittal alignment parameters, and adequately powered multivariable analyses are needed to determine the independent contribution of distal fixation strategy in optimizing long-term construct survival and patient outcomes.

## Data Availability

The datasets used and analyzed during the current study are available from the corresponding author upon reasonable request.

## Abbreviations

ALIF: anterior lumbar interbody fusion
ASA: American Society of Anesthesiologists
BMI: body mass index
CI: confidence interval
IS: iliac screw
ODI: Oswestry Disability Index
OR: odds ratio
PI–LL: pelvic incidence–lumbar lordosis mismatch
PT: pelvic tilt
S2AI: S2-alar-iliac
SD: standard deviation
SS: sacral slope
SVA: sagittal vertical axis
TLIF: transforaminal lumbar interbody fusion
